# Two Decades of Melioidosis in India: A Comprehensive Epidemiological Review

**DOI:** 10.3390/pathogens14040379

**Published:** 2025-04-14

**Authors:** Sriram Kannan, Suchita Singh, Venkat Abhiram Earny, Soumi Chowdhury, Mohammed Ashiq, Vandana Kalwaje Eshwara, Chiranjay Mukhopadhyay, Harpreet Kaur

**Affiliations:** 1 Division of Communicable Diseases, ICMR, Indian Council of Medical Research, New Delhi 110029, India; sriram.ecdicmr@gmail.com (S.K.); suchitaasingh@gmail.com (S.S.); 2 Department of Microbiology, Kasturba Medical College, Manipal Academy of Higher Education, Manipal 576104, Indiasoumi.sia1995@gmail.com (S.C.); drmohammedashiqs@gmail.com (M.A.); vandana.ke@manipal.edu (V.K.E.); 3 Center for Emerging and Tropical Diseases, Kasturba Medical College, Manipal Academy of Higher Education, Manipal 576104, India

**Keywords:** melioidosis, tropical disease, diabetes, poverty, hot spots, Southeast Asia, India

## Abstract

Melioidosis, caused by *Burkholderia pseudomallei*, is a potentially fatal infection, particularly affecting individuals with chronic conditions such as diabetes or kidney or liver diseases. This review examines melioidosis in India over the past two decades, focusing on its prevalence, risk factors and clinical manifestations. A PubMed search (2000–2024) identified a rise in melioidosis publications, with most from Southern India, followed by Eastern India, and an increase post-2019. Eight studies from 2010–2022 identified fever (86%), cough (26%) and joint pain (23%) as the most common symptoms, while diabetes (75%), alcohol abuse (19%) and cancer (6%) were primary predisposing factors. Severe clinical manifestations were also observed, including bacteremia (50%), pneumonia (37%) and splenic abscess (18%). Although environmental exposure risks were not significantly high, individuals with diabetes or chronic kidney disease, particularly those working in high-risk environments, were more likely to contract melioidosis. Cryptic environmental factors that might bridge known epidemiological risk factors are also addressed. The review emphasizes the increasing awareness and research in clinical epidemiology and also highlights a gap in studies on antimicrobial treatments, vaccines and environmental surveillance. Targeted interventions in diabetes and poverty hotspots could help control the disease more effectively.

## 1. Introduction

Melioidosis is predominantly a tropical disease, prevalent especially in Southeast Asia and Northern Australia, caused by the bacterium *Burkholderia pseudomallei*. This soil saprophyte is classified as a Tier 1 select agent due to its potential as a bio threat. *B. pseudomallei* is typically found in soil and water, where it can infect humans and animals through the inhalation of contaminated dust or water droplets, ingestion of tainted water or skin abrasions, primarily via contact with contaminated soil [1]. However, limited awareness of the disease among clinicians and microbiologists often leads to misdiagnosis and inappropriate treatment.

As an emerging infectious disease, melioidosis contributes substantially to the global disease burden, with an estimated 4.64 million disability-adjusted life years (DALYs) lost annually [2,3]. The first clinical case of melioidosis was reported in Myanmar in 1911. Although it is believed that *B. pseudomallei* may have originated in Australia, it is thought to have spread to Asia and Southeast Asia between 16,000 and 225,000 years ago, possibly via migrating populations, trade routes and animals [4]. The pathogen may have been introduced to Madagascar around 2000 years ago and to the Americas between 1650 and 1850 AD [4].

### 1.1. Environmental Presence and Studies in South Asia

*B. pseudomallei* thrives in soil at depths greater than a foot, often rising to the upper surface during the monsoon season or through human activities such as agriculture (working in rice fields), construction and sports. Studies suggest that this bacterium is commonly associated with regions characterized by high rainfall, temperature and specific soil types, such as anthrasol and acrisol soils. Research by Oxford University predicted that *B. pseudomallei* would remain ubiquitous throughout the tropics, with high-risk zones extending not only across South Asia but spreading to parts of Australia, South America and West Sub-Saharan Africa [5]. This study used a 5 km × 5 km gridded model that incorporated soil characteristics from the Harmonized World Soil Database, as well as climatic conditions and moisture levels. Additionally, it has been shown that elevated temperatures (37–40 °C) and rainfall facilitate the replication and spread of *B. pseudomallei* through soil [5,6].

While melioidosis was first recognized for its high endemicity in Thailand and Northern Australia, it has since been reported in areas such as China, South America and parts of Africa and Asia [7]. The modeling study by Oxford University estimated that there are 165,000 annual cases of melioidosis globally, with 89,000 deaths, and predicted that India has a significant burden of the disease, with 52,000 cases and 31,425 deaths each year. However, between 1991 and 2016, only 583 cases were reported in India, with 231 cases originating from Manipal between 2006 and 2016. This discrepancy could be attributed to poor diagnostic facilities, lack of awareness and unidentified hotspots in certain regions [2].

### 1.2. Environmental Factors and Risk Groups

The environmental factors contributing to melioidosis include monsoons, inhalation of aerosolized bacteria, skin abrasions, ingestion of contaminated water, occupational hazards and physical injuries such as thorn pricks [8]. Studies indicate that male agricultural workers are particularly vulnerable to melioidosis, with fever being the most common symptom. Musculoskeletal and lung involvement are also frequently observed, with the latter being the most common target organ [9]. Other organs affected by the disease include bones, prostate, blood, liver, skin, soft tissue, spleen, aorta, brain, bone marrow and kidneys [10]. The disease may also manifest as a cutaneous form, either due to a primary skin infection or dissemination from another infected organ. Skin lesions range from papules and nodules to pustules and ulcers, resembling symptoms seen in other infectious diseases such as tuberculosis, plague, anthrax, cat scratch disease and sporotrichosis [10]. Although diabetes, poverty, alcoholism and agricultural activities are known risk factors for melioidosis, a study published in 2024 found that 20–36% of cases had no identifiable predisposing factors [11]. In contrast, another study reported diabetes as a risk factor in 81.6% of cases [12]. Thus, environmental factors and lack of awareness, influenced by poverty and poor sanitation, could contribute significantly to melioidosis risk. 

Additionally, a study on genetic susceptibility to pneumonia during COVID-19 categorized India as having a moderate genetic risk. Taking this into consideration, it could be ascertained that environmental factors and lack of awareness constitute risk factors for melioidosis. Genetic studies related to melioidosis, similar to those in Thailand, could be useful for India [13].

Melioidosis primarily affects people in rural areas with close ties to agriculture and livestock and given that its risk factors encompass environmental, animal and anthropogenic activities a “One Health” approach has been suggested for melioidosis management. Integrating veterinary, medical and environmental efforts can enhance surveillance, improve diagnostic capabilities and promote awareness [14]. By educating communities on prevention strategies such as proper hygiene and safe agricultural practices, the One Health framework aims to reduce transmission rates. For instance, Malaysia has implemented action plans through collaborations between various ministries, including those focused on agriculture, veterinary services and medical research [15]. Thus, collaborative research and policymaking are crucial for effective management and control of melioidosis, ultimately protecting vulnerable populations and ecosystems in India.

- Cryptic environmental factors bridging risk factors of melioidosis:
  ○ Blow flies: Melioidosis is always correlated to monsoon season and rice farming, but there could be cryptic environmental factors associated with soil and rain. One such factor could be insects associated with monsoon. In one study from Malaysia, blow flies have been reported to harbor *B. pseudomallei* [16]. These flies exhibit seasonal variation and are attracted to fish processing [17]. Integrated fish/prawn and paddy cultivation could possibly attract blow flies as well as increase chances of skin abrasion in rice farmers owing to fish or prawn exoskeletons, thus increasing the chances of *B. pseudomallei* infection. Thus, there could be cryptic epidemiological factors that could influence risk factors for melioidosis.
  ○ Wound myiasis: Melioidosis is associated with poverty, alcoholism, diabetes, and agricultural occupation, but the cryptic environmental factor that could be associated with all these four could include blow flies like *C. megacephalus* that can cause wound myiasis [18]. Myiasis is known to have risk factors like alcoholism, malnutrition, poor hygiene, and diabetes [19]. In general, *C. megacephalus* flies are attracted towards human feces, putrefying substances, and mainly fish products [20].
  ○ Erythritol-enriched niche—natural and manmade: It is known that erythritol present in the placenta of animals favors pathogenesis in Brucella infections, leading to abortion in animals [21]. Erythritol, also known to favor the growth of *B. pseudomallei* in culture media, could possibly indicate that domestic animals with brucellosis might also have the potential to harbor *B. pseudomalli.* Further, India ranks high in stray cattle with Brucellosis [22], and road or rail accidents involving animals with improper carcass disposal could enrich pathogens locally. Furthermore, since erythritol is used as insecticide [23], it might influence the environmental load of *B. pseudomallei.*

Further, recent studies have also shown that individuals with diabetes and those exposed to environmental contaminants have a higher likelihood of contracting melioidosis, with poor drinking water quality being an additional risk factor. In particular, the use of chlorinated and boiled drinking water is emphasized to reduce the risk [24]. Melioidosis is endemic to the neighboring countries of India and Cambodia, but Cambodia reports relatively few cases, likely due to limited diagnostic facilities and a lack of awareness, a situation that could also be true for India. In Cambodia, environmental factors such as rainfall, wind speed, humidity and low visibility were found to be significantly associated with melioidosis incidence [25].

Wind speed was identified as a risk factor for melioidosis in Cambodia [26]. The odds ratio for males compared to females was 0.74/0.73 for medium- and high-speed winds, indicating males to be more prone to melioidosis through wind exposure. Children had a higher odds ratio (1.44/1.13) of contracting the disease in medium- and high-speed winds, with the former posing a greater risk. Rice farmers had an increased odds ratio (1.31/1.36) of acquiring melioidosis in medium- and high-speed winds compared to other professions. Blood glucose levels >150 mg/dL were associated with an odds ratio of 1.07/1.09 for medium- and high-speed winds. The history of melioidosis showed a lower odds ratio (0.87/1.06) for medium- and high-speed winds. Notably, individuals with diabetes were more likely to develop melioidosis in high-speed winds (>13 km/h), while those without diabetes were more susceptible in medium-speed winds (10–13 km/h). Additionally, individuals with disseminated infections had a higher odds ratio (1.46/1.88) in medium- and high-speed winds, while those with lung infections had an odds ratio of 1.34/1.93. Those with skin or soft tissue infections had an odds ratio of 1.00 in medium-speed winds and 0.68 in high-speed winds [26].

A study from South India highlighted that the prevalence of diabetes among patients with melioidosis was higher in India than in Northern Australia, Thailand and Malaysia [27]. While environmental exposure and mortality rates were comparatively lower in India, recent studies indicate that melioidosis can affect people from various occupations, not limited to rural agricultural workers. Occupations related to soil exposure include farmers, lumberjacks, landscapers, agriculturists, construction workers, renovators and military personnel, while water exposure was linked to fishermen, sailors and shipyard workers. The risk of infection post-rainfall was found to be 79%, underlining the association of water and rainfall with melioidosis outbreaks. Interestingly, it has been noted that not only rural areas associated with agriculture but also urban slums and villages could harbor *B. pseudomallei*, the pathogen responsible for melioidosis, as demonstrated in a study from New Delhi, where the bacterium was found in urban market areas [28]. Additionally, studies have shown that even 1000 ppm chlorine was found to be ineffective in eliminating *B. pseudomallei* from water sources, thus reinforcing the need for better environmental management in endemic regions [29].

### 1.3. The Importance of Melioidosis in India, Clinical Challenges and the Need for Awareness

Despite the growing recognition of melioidosis, there is still no formal surveillance for the disease in South Asia, particularly in India, where it is often misdiagnosed as tuberculosis or common pneumonia (CDC, Melioidosis Symptoms) [30]. India, often referred to as the diabetic capital of the world, has 77 million people affected by diabetes, with a prevalence rate of 8.9% [31] The high prevalence of diabetes, combined with factors such as state-wise average annual rainfall and widespread paddy cultivation, creates an environment conducive to the growth of *B. pseudomallei*. Given these factors, India has a high potential to become an endemic country for the disease, underscoring the urgent need for increased awareness, surveillance, and research to mitigate the growing public health threat it presents [32].

Further, melioidosis also presents significant challenges for clinicians due to its diverse clinical manifestations, including bacteremia, sepsis, pneumonia, skin and soft tissue infections, abscesses (intra-abdominal, lung, prostate, renal, parotid, brain, tubo-ovarian), osteomyelitis, septic arthritis and neurological involvement [33]. A 10-year study identified several key predictors of in-hospital mortality in melioidosis patients including age (OR 1.01), male gender (OR 0.47), diabetes mellitus (OR 0.89), hypertension (OR 1.48), chronic kidney disease (CKD) (OR 0.71), chronic liver disease (CLD) (OR 3.81), acute kidney injury (AKI) (OR 7.63), and hyponatremia (OR 3.05) [34]. 

Thailand and Malaysia documented high exposure to soil and water, diabetes mellitus, hematologic or solid tumors, renal disease, thalassemia, open wounds, consumption of food contaminated with soil, rain exposure, drinking untreated water, aerosol inhalation, smoking, and steroid use as risk factors for melioidosis [24].

Also, In Indonesia, reported diabetes mellitus (56%), CKD (19%), chronic liver disease (7%), malignancy (7%), alcohol abuse (4%), chronic lung disease (4%) as known risk factors for melioidosis but no predisposing factors in 26% of cases [35]. Whereas, in Thailand, another study of 1121 melioidosis patients identified diabetes (71%), hypertension (31%), dyslipidemia (8%), CKD (16%), smoking (35%), heart disease (5%), stroke (3%), lung disease (12%), tuberculosis (8%), alcohol use disorder (5%), and HIV (1%) as risk factors [35]. 

Additionally, while in India, common risk factors for melioidosis include poorly controlled diabetes, chronic kidney disease (CKD) and chronic alcoholism. In contrast, a study from Thailand identified additional risk factors such as dyslipidemia, chronic corticosteroid therapy and gout, which were not observed in Indian studies [36]. In this context, a comprehensive review from Australia [37] involving 31 melioidosis cases with cardiac involvement, including patients from multiple countries (including 4 from Thailand, 5 from Malaysia, 3 from Vietnam, 3 from Singapore, 9 from India, 2 from Sri Lanka, 2 from China, 1 from Australia, 1 from Panama, and 1 from Puerto Rico), revealed that 95% of cases were males, with an average age of 58 years. Common risk factors included soil exposure (49%), water exposure (11%), travel (5%), diabetes (38%), coronary artery disease (8%), airways disease (3%), smoking (21%), hypertension (8%), alcohol consumption (8%), malignancy (5%), chronic liver disease (3%), and CKD (5%).However, the true prevalence of melioidosis remains largely unknown.

In children, melioidosis typically presents as parotid abscess and cutaneous infections, which occur four times more frequently in children than in adults. In contrast, adults are more likely to develop pneumonia, which is three times more common in adults than in children [38]. Melioidosis is commonly present with community-acquired pneumonia with or without septicemia, which is considered to be highly fatal [39].

A case study involving two brothers from Kerala, India, exemplifies the variable clinical presentation of melioidosis. The two brothers tested positive for melioidosis. One developed pneumonia, ARDS and septic shock, while the other presented with fever, sore throat and lymphadenopathy. The latter survived, whereas the former succumbed to the disease, highlighting thereby both clinical variability and the potential for misdiagnosis of the disease [40].Timely detection and treatment with appropriate antibiotics are crucial for reducing mortality.

### 1.4. Diagnostic Challenges

A review of diagnostic practices from Southeast Asian countries including Thailand and Malaysia revealed a variety of assays used for melioidosis detection such as blood, sputum, synovial, pus, aspirate and urine cultures, along with multiplex PCR, VITEK2, three-disk test, and AMD-LFA. For culture-negative tuberculosis patients, antibody detection methods, such as indirect hemagglutination (IHA), immunofluorescence assays (IFA) for IgM and IgG, and serological tests were commonly employed. Tsunami survivors were tested using IHA antibody detection [24]. For febrile patients, ELISA using OPS and Hcp1 was used. For military personnel, ELISA with exotoxin and whole-cell antigen was used [41]. Notably, antibody detection plays a significant role in identifying patients with suspected melioidosis, especially in those presenting with fever, sepsis of unknown origin and as part of post-disaster screening efforts.

The gold standard for diagnosis remains the culture of *B. pseudomallei* from clinical specimens. While culture-based diagnostic methods are reliable, they are time consuming and require a BSL-II facility for processing clinical materials of human and animal origin, with further BSL3 containment needed for handling cultures and conducting experimental studies. Additionally, their sensitivity can be variable depending on specimen type and bacterial load, limiting their utility in resource-constrained settings [42], due to the intermittent bacteremia observed in melioidosis [43]. Furthermore, they may not always yield positive results due to antibiotic therapy, making PCR-based methods an attractive alternative for rapid diagnosis. Such molecular assays, including real-time PCR targeting *B. pseudomallei*-specific genes, offer higher sensitivity and specificity but remain expensive and largely unavailable in endemic rural areas [44,45]. However, the accuracy, sensitivity and specificity of PCR-based diagnostics still require further investigation to ensure they outperform traditional culture methods in varied clinical settings. A summary of the comparison of PCR-based diagnostics, culture and serological methods for the detection of melioidosis across various studies is provided in Table 1 and Table 2, which compare the various methods employed, the target genes used for PCR assays, the number and types of clinical or environmental samples analyzed, and the reported sensitivity, specificity, turnaround time of each method across various studies. Commonly targeted genes include TTSS1, wcbG and 16S rRNA, with sensitivity and specificity varying based on the diagnostic technique, sample type and study design.

The tables outline the advantages and limitations of each method, offering insights into the appropriate choice of diagnostic tools depending on the clinical setting and available resources, highlighting that culture remains the gold standard for specificity, while PCR-based methods offer higher sensitivity and faster turnaround times. Furthermore, the lateral flow immunoassays, which detect the capsular polysaccharides, provide a rapid point-of-care option and show promise for rapid bedside diagnosis, but with lower sensitivity in non-bacteremic cases; therefore, they require further validation for routine clinical use [46].

On the other hand, serological tests such as the indirect hemagglutination assay (IHA) have variable sensitivity and specificity, with high background seropositivity in endemic regions, making it difficult to differentiate past exposure from an active infection [47,48,49,50].

As melioidosis is frequently misdiagnosed as tuberculosis or pneumonia due to its diverse clinical manifestations and overlapping symptoms with other bacterial infections, diagnostic methods must be carefully considered. Since melioidosis can mimic tuberculosis, patients with negative tuberculosis tests (e.g., AFB or GeneXpert) should be screened for melioidosis, especially those suspected of having extra-pulmonary tuberculosis [51].

**Table 1:** Comparison of diagnostic techniques for detection of *Burkholderia pseudomallei* across various studies.

Sl. No.	References	Diagnostic Techniques	Targets	Numbers of Samples	Types of Samples	Sensitivity (%)	Specificity (%)
1	[27]	PCR	*TTSS1* gene	525	Sputum, wound swab, pus, BAL, tissue, ET aspirate	99.30%	-
2	[52]	PCR	*16s rRNA* gene	29	Blood	47.37%	100%
3	[53]	PCR	*TTSS1* gene	71	Blood	100%	100%
4	[54]	real time PCR	*TTSS1* gene	209	Sputum	100%	100%
5	[55]	Culture (PEG-DOC solution used)		30	Soil	100%	
6	[56]	real time PCR	*TTSS1* gene	399	Blood, sputum, urine, pus, tissue	49.48%	98.05%
7	[57]	PCR	*16s rRNA* gene	846	Blood, sputum, urine, pus, tissue	50.86%	99.04%
8	[58]	PCR		99	Blood	38.89%	93.83%
9	[59]	PCR	*TTSS1* gene	93	Blood, sputum, urine, pus, tissue	35.71%	100%
10	[60]	PCR	*TTSS1* gene	846	Blood, sputum, urine, pus, tissue	30.17%	98.49%
11	[61]	PCR-LFD	*wcbG* gene	43	Blood	100%	100%
12	[62]	Real-time PCR	-	28	Blood	72%	82%
13	[62]	Real-time PCR	-	17	Blood	58%	88%

**Table 2:** Comparison of PCR-based diagnostics, culture, and serological methods for melioidosis detection.

Diagnostic Method	Sensitivity	Specificity	Turnaround Time	Advantages	Limitations	Reference
Culture (Gold Standard)	~60–80% (varies by specimen type)	100%	2–7 days	Definitive diagnosis, confirms viable *B. pseudomallei*	Slow, requires BSL-3 lab, sensitivity affected by prior antibiotic use	[42]
Blood Culture	~60%	100%	2–5 days	Useful for bacteremic cases	Low sensitivity, often negative in non-septicemic patients	[43]
PCR (e.g., real-time PCR, 16S rRNA, TTS1 target)	~85–95%	~95–99%	4–6 h	Rapid, high sensitivity, useful in early diagnosis	Expensive, requires specialized equipment, not widely available in endemic areas	[44]
Lateral Flow Immunoassay (for capsular polysaccharide antigen detection)	~80–90%	~95%	15–30 min	Rapid, bedside diagnosis, does not require specialized lab	May have reduced sensitivity in non-bacteremic cases	[45]
Indirect Hemagglutination (IHA)	51–95% (varies by region and infection stage)	74–97% (depends on assay quality and antigens used)	1–2 h	Rapid, relatively easy to perform, cost-effective, useful for screening	False positives, cross-reactivity with other infections, does not distinguish between active or past infection	[48,49,50]

In conclusion, melioidosis remains a significant but under-recognized infectious disease, with increasing global awareness needed, particularly in regions like India where its potential for endemism is high. Early detection, appropriate treatment and further research into environmental and clinical risk factors, along with the development of more effective diagnostic methods, are critical for reducing its mortality, improving clinical outcomes and reducing the global burden of melioidosis.

## 2. Materials and Methods

**Figure 1:**
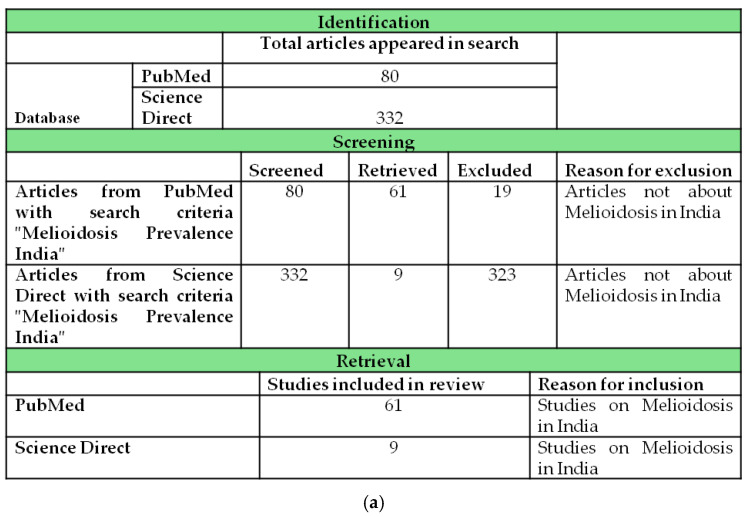

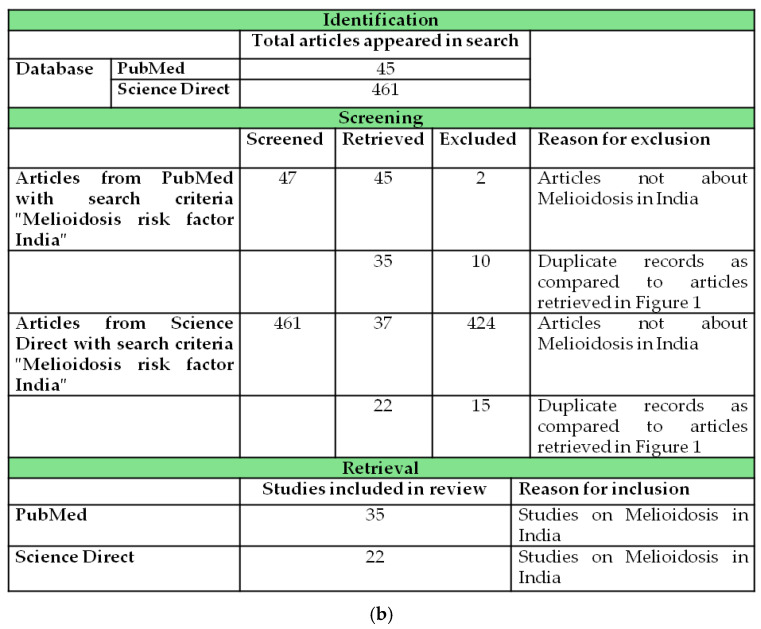
Selection of articles from PubMed and Science Direct: (**a**) with search words “melioidosis prevalence India”; (**b**) with search words “melioidosis risk factor India”. The articles with no relevance to melioidosis in India were excluded.

This study comprehensively reviews literature obtained from PubMed (total articles retrieved: 96) and Science Direct (total articles retrieved: 31), focusing on two specific search criteria, “melioidosis prevalence India” and “melioidosis risk factor India”, as shown in Figure 1a,b. The search was conducted for articles published during the last two decades, covering the years 2000–2024, and grouped into four distinct time periods, 2024–2019, 2019–2014, 2014–2009, and 2008–2000, as detailed in Supplementary Table S1 [1,7,27,32,40,63,64,65,66,67,68,69,70,71,72,73,74,75,76,77,78,79,80,81,82,83,84,85,86,87,88,89,90,91,92,93,94,95,96,97,98,99,100,101,102,103,104,105,106,107,108,109,110,111,112,113,114,115,116,117,118,119,120,121,122,123,124,125,126] and Table S2 [11,12,27,33,49,66,67,68,69,127,128,129,130,131,132,133,134,135,136,137,138,139,140,141,142,143,144,145,146,147,148,149,150,151,152,153,154,155,156,157,158,159,160,161,162,163,164,165,166,167,168,169,170,171]. Relevant articles were selected based on their relevance to the epidemiology and risk factors associated with melioidosis in India. The retrieved studies were analyzed for key findings regarding the prevalence of melioidosis, the identification of risk factors, and trends over time, aiming to provide a comprehensive overview of the disease’s impact in India during the specified periods.

## 3. Results

### 3.1. Comparison of Publications on Melioidosis over the Years

The number of publications on melioidosis in India has shown a significant increase over the past two decades. Most research publications has concentrated in South and Southwest India. However, by 2024, the distribution of publications had broadened. There has been a notable rise in publications from both South India and other regions, such as the east and other parts of the country. This growing trend indicates an increasing awareness of melioidosis across India, although the number of publications remains comparatively low in Central, North and Northeast India, thus suggesting the need for further research and awareness efforts required in these regions, where melioidosis remains under-explored. Individual publications with the criteria published are detailed in Supplementary Tables S1 and S2.

**Figure 2:**
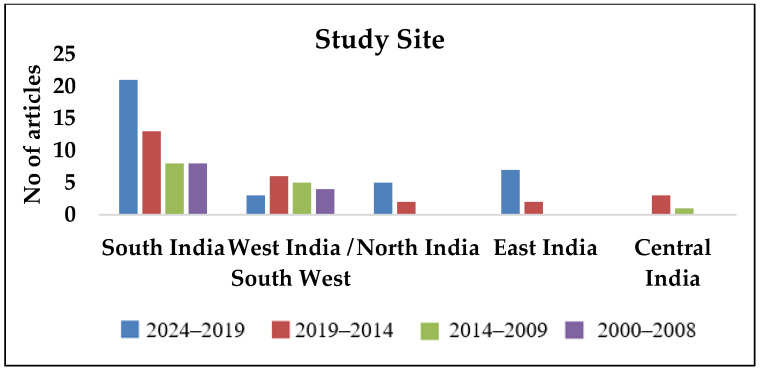
Year-wise segregation of publications based on study site.

**Figure 3:**
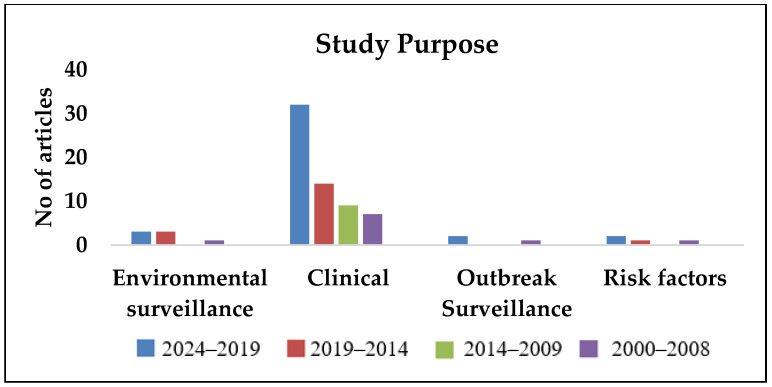
Year-wise segregation of publications based on study purpose.

**Figure 4:**
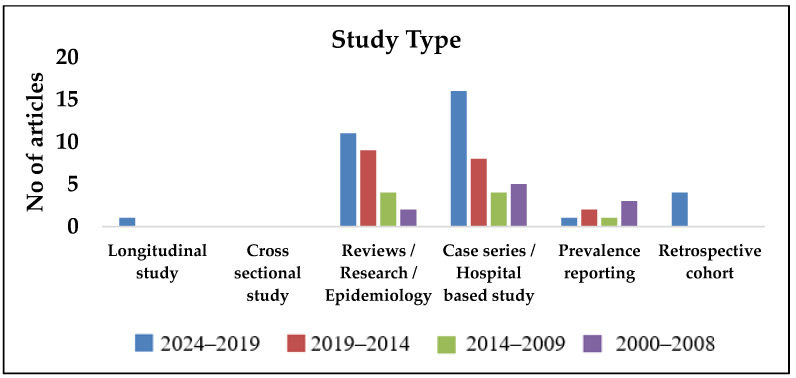
Year-wise segregation of publications based on study type.

**Figure 5:**
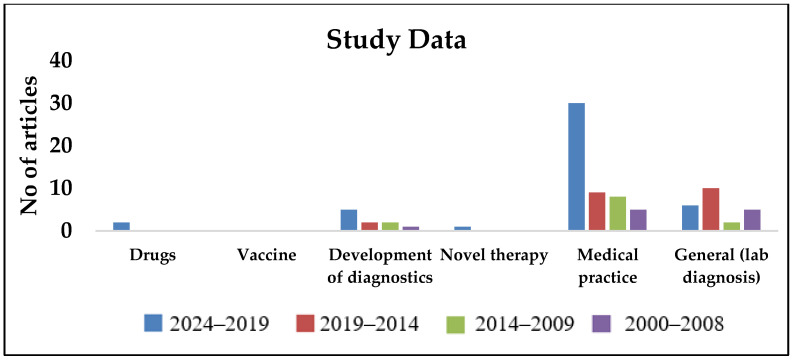
Year-wise segregation of publications based on study data.

**Figure 6:**
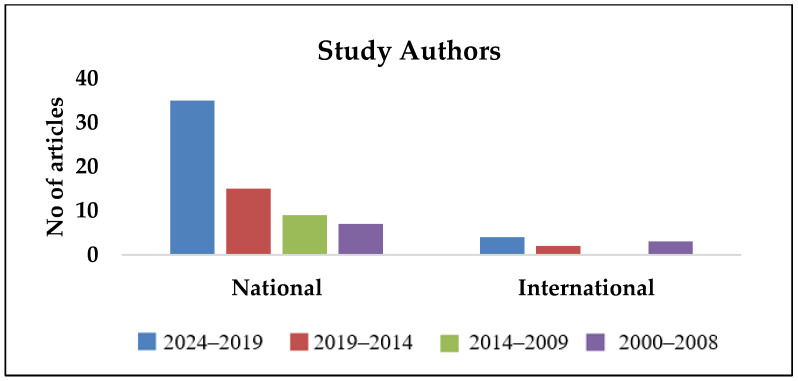
Year-wise segregation of publications based on study authors.

As observed in Figure 2, Figure 3, Figure 4, Figure 5 and Figure 6, most publications have predominantly been focused on clinical and epidemiological data, and publications concerning environmental surveillance have been relatively fewer. Similarly, studies on outbreak surveillance and risk factors were limited to 1–2 articles in the past decade. This highlights the need for further work in the area of environmental and outbreak surveillance, especially if melioidosis is designated a notifiable disease in the future in India. In the past five years, the publications have mainly focused around general clinical and laboratory data, medical practices, novel therapies, developmental diagnostics and drug research.

### 3.2. Outcome of Pooling Study Data on Symptoms and Risk Factors of Melioidosis

Data from nine studies, including publications from 2022, 2021, 2019, 2012 and 2010, were pooled to analyze the symptoms and risk factors associated with melioidosis in India as indicated in Table 3.It was observed that fever was the most common symptom, affecting 86% (SD = 12%) of patients, followed by cough (26%, SD = 17%) and joint pain (23%, SD = 21%). The most prevalent predisposing condition was diabetes (75%, SD = 9%), followed by alcohol abuse (19%, SD = 9%) and cancer (6%, SD = 1%).

The clinical presentation of melioidosis in India also revealed that bacteremia was observed in 50% (SD = 38%) of cases, with skin and soft tissue involvement in 16% (SD = 10%), pneumonia in 37% (SD = 23%), and splenic abscess in 18% (SD = 16%) of patients.

### 3.3. Treatment of Melioidosis in India

A study from Eastern India, involving isolates from pyogenic lesions, found that *B. pseudomallei* accounted for 25% of isolates. Among these, 35% were laborers, 6% military personnel, 6% drivers, 6% farmers, 24% homemakers, 15% students, 6% office workers, and 3% teachers. The risk factors included diabetes (29%), diabetes with alcoholism (15%), and alcoholism alone (12%). The initial treatment regimen primarily involved ceftazidime (82%), followed by meropenem (12%) and imipenem (3%) [64]. For eradication, co-trimoxazole was used in 88% of patients, doxycycline in 6%, and amoxicillin-clavulanate in 3%. However, carbapenems are less commonly used in India due to economic constraints, with ceftazidime being the preferred option [36].Emerging resistance to these antibiotics is complicating treatment. A significant case from Mumbai highlighted a *B. pseudomallei* isolate resistant to ceftazidime but susceptible to carbapenems and co-trimoxazole [162]. The patient was successfully treated with meropenem and co-trimoxazole for eight weeks, followed by continued co-trimoxazole therapy for eight months, demonstrating the potential of carbapenems in managing melioidosis in India. Recent case series from India indicate that meropenem or ceftazidime are commonly used to treat melioidosis, with some patients requiring intensive care, though resistance patterns were not specified [172]. The emergence of ceftazidime resistance and the variable efficacy of meropenem underscore the importance of ongoing surveillance to guide treatment decisions. While carbapenems offer an alternative treatment option, their use must be tailored based on local resistance patterns. As we do not have enough data at present on the prevalence and clinical significance of AMR in melioidosis, especially on the first-line antibiotics like ceftazidime and carbapenem, we can acknowledge this as a research gap. Therefore, continuous monitoring of the evolution of antibiotic resistance is essential to ensure the effectiveness of treatment strategies and improve patient outcomes in melioidosis.

### 3.4. Mapping of Indian States with Diabetes as a Melioidosis Risk Factor

**Figure 7:**
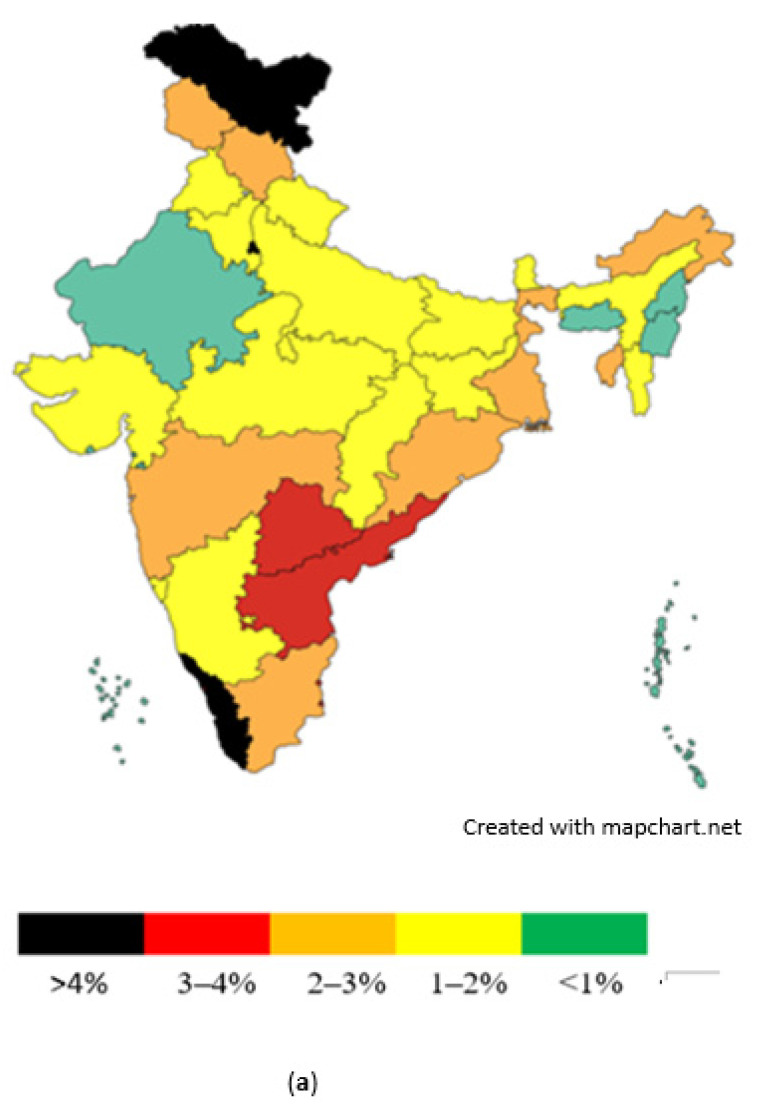

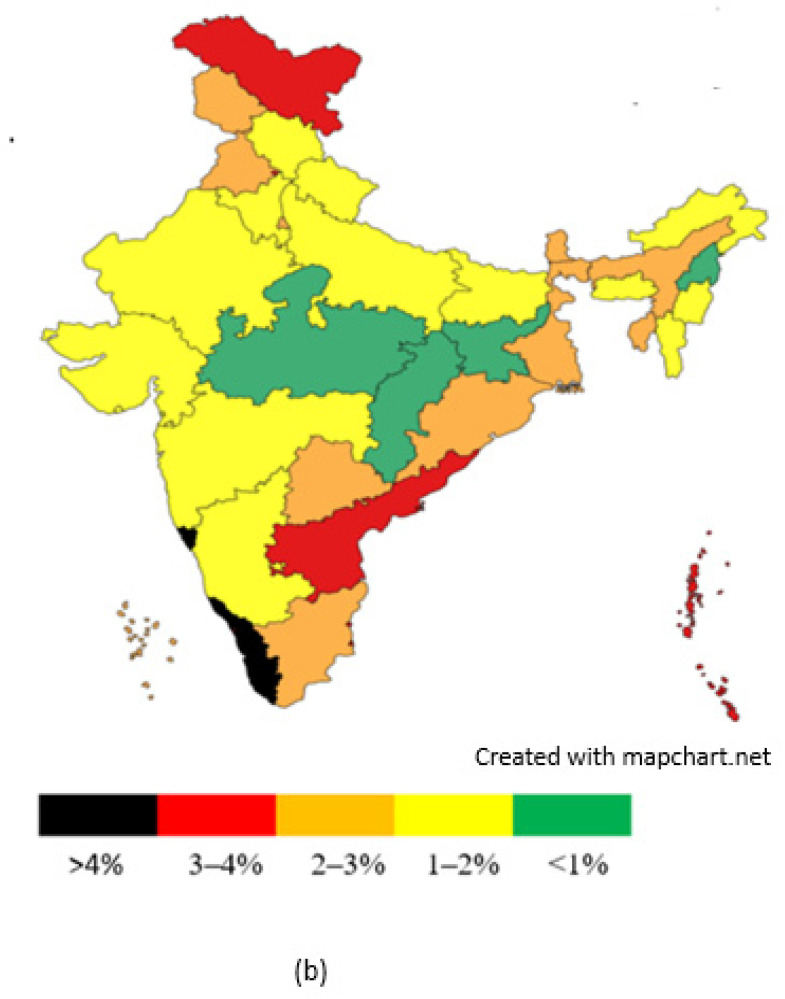

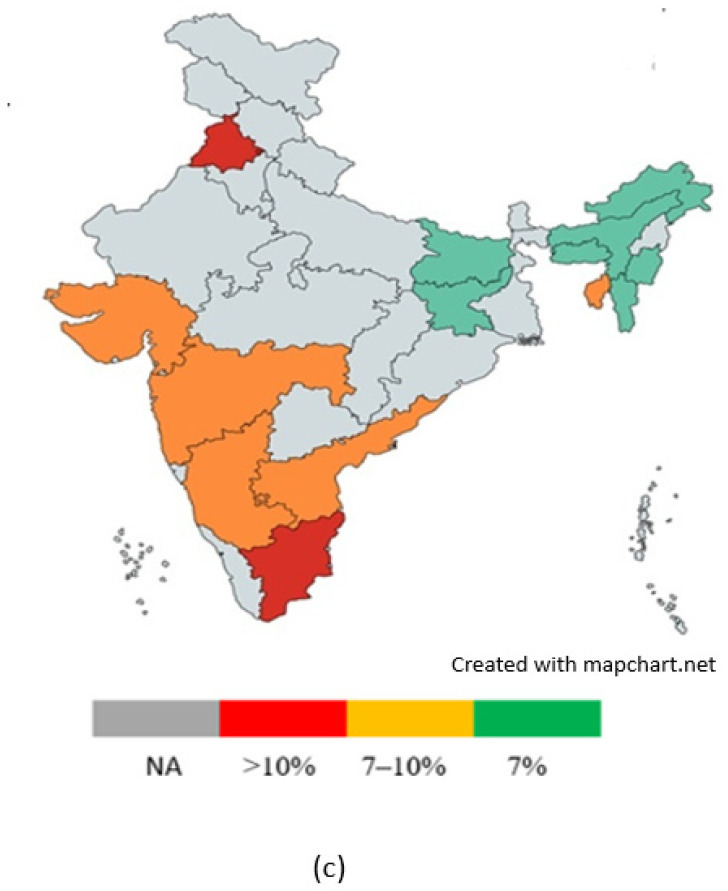

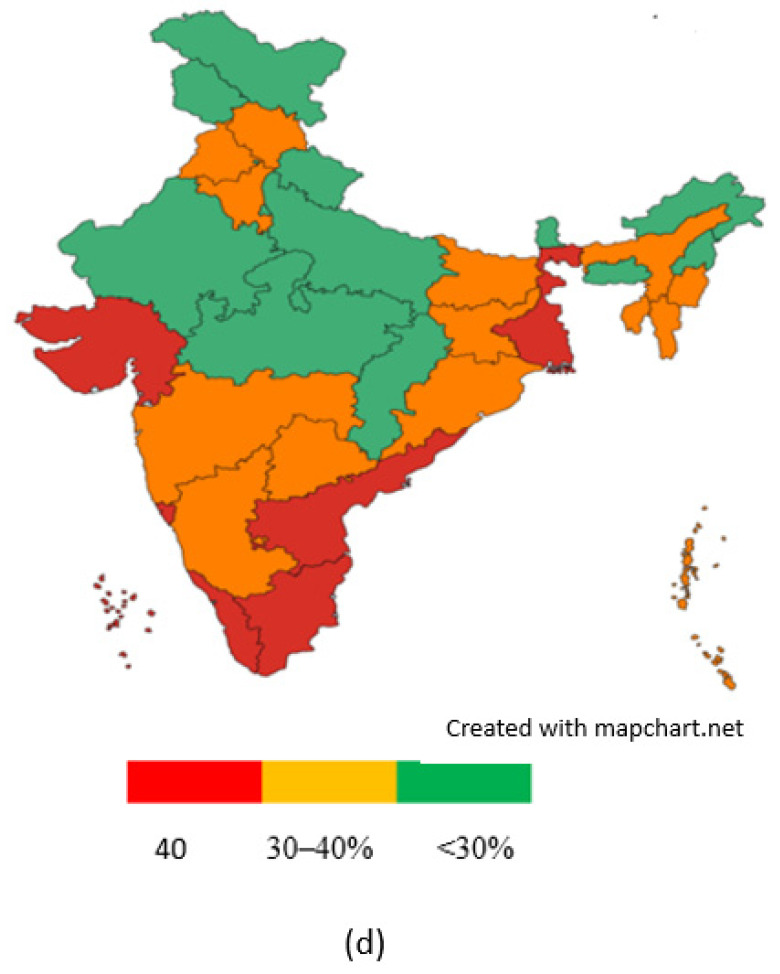

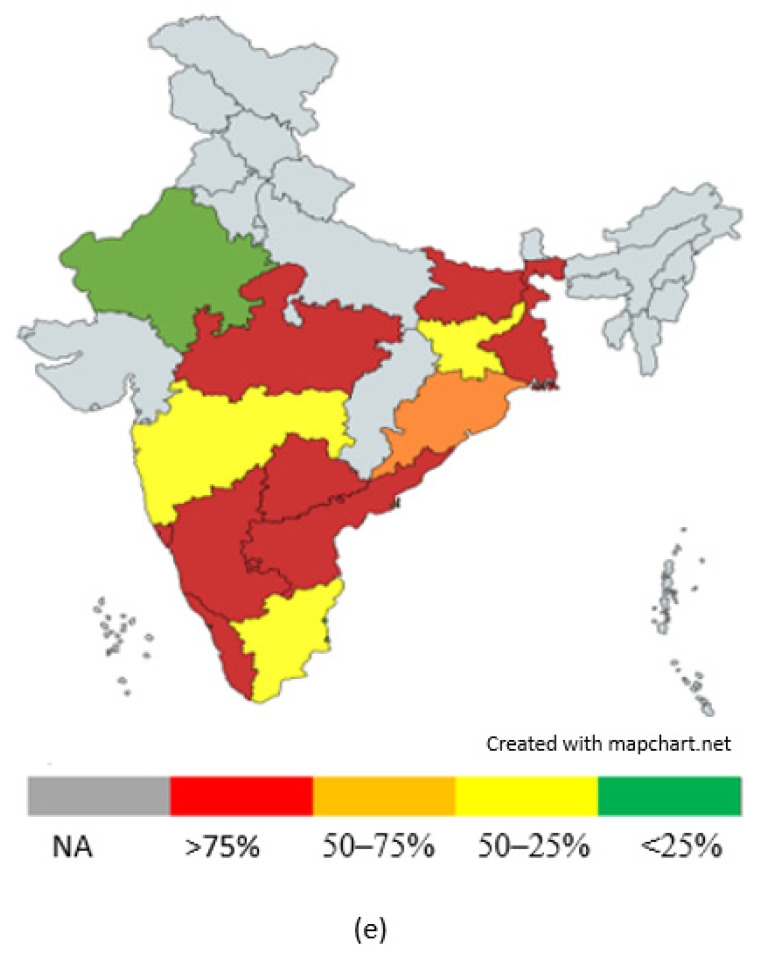
(**a**) Prevalence of diabetes in male population of Indian states (NFHS-5 data)—2019–2021. (**b**) Prevalence of diabetes in female population of Indian States (NFHS-5 data)—2019–2021. (**c**) Prevalence of diabetes in selected states [173]. The states that are shaded grey were not included in the study (NA); States shaded in red indicate high prevalence (10%); States shaded in orange indicate moderate prevalence (7–10%); and states shaded in green indicate low prevalence (>7%). (**d**) Proportion of households with one diabetic member across Indian states [174]. The states shaded in red are >40%. States shaded in orange are 30–40%, and states shaded in green are <30%. (**e**) Diabetes cases with melioidosis in selected Indian states (1991–2018) [172].

Taking diabetes as a risk factor for melioidosis, as shown in Figure 7a,b, it can be hypothesized that states with higher diabetes prevalence may have a higher prevalence of melioidosis. The following states are suggested to have a higher risk of melioidosis: Gujarat, Kerala, West Bengal, Tamil Nadu, Andhra Pradesh, and Goa.

According to the NFHS-5 data (2019–2021) for the male diabetic population (age 15–54 years), states with a very high prevalence of diabetes include Kerala, Delhi, and Ladakh, followed by Andhra Pradesh and Telangana, as shown in Figure 7a. States with high diabetes prevalence include Maharashtra, Tamil Nadu, Odisha, West Bengal, Arunachal Pradesh, Tripura, Jammu and Kashmir, and Himachal Pradesh. States with a moderate level of diabetes prevalence include Goa, Karnataka, Chhattisgarh, Madhya Pradesh, Jharkhand, Punjab, Haryana, Uttarakhand, Bihar, Sikkim, Assam and Mizoram. States with a low prevalence of diabetes include Rajasthan, Meghalaya, Nagaland and Manipur.

In terms of the female diabetic population (age 15–49 years), the NFHS-5 data (2019–2021) reveal that Kerala and Goa have the highest rates, followed by Andhra Pradesh and Ladakh, as shown in Figure 7b. Other states with significant female diabetes prevalence include Tamil Nadu, Punjab, Jammu and Kashmir, Telangana, Odisha, West Bengal, Assam, Sikkim, and Tripura. States with moderate female diabetes prevalence include Karnataka, Maharashtra, Gujarat, Rajasthan, Haryana, Uttar Pradesh, Bihar, Arunachal Pradesh, Meghalaya, Mizoram, and Manipur. The lowest female diabetes prevalence is seen in Madhya Pradesh, Chhattisgarh, and Jharkhand.

Two additional studies [173,174] provide further data on diabetes prevalence in India, as shown in Figure 7c,d. A study conducted at Kasturba Medical College, Manipal [172], documented a 20-year dataset on the prevalence of melioidosis across various Indian states (Figure 7e). Karnataka reported the highest number of melioidosis cases, with 499 cases and an 8% mortality rate. Among these cases, 78% were diabetic, and the male-to-female ratio was 3:1. Tamil Nadu reported 210 melioidosis cases (22% mortality), with 57% of cases being diabetic and a male-to-female ratio of 3:1. Kerala reported 58 cases (10% mortality), with 56% of cases being diabetic. Puducherry reported 79 cases (18% mortality), with 26% of cases being diabetic and a male-to-female ratio of 3:1. Telangana had 36 melioidosis cases (11% mortality), with 77% of cases being diabetic and a male-to-female ratio of 2:1. Maharashtra reported 10 cases (40% mortality), with 60% of cases being diabetic and a 9:1 male-to-female ratio. Goa had 7 cases, with 85% being diabetic, and a male-to-female ratio of 5:0. Bihar reported 5 cases, all diabetic, with a male-to-female ratio of 4:1. Jharkhand reported 2 male cases, while Madhya Pradesh, Andhra Pradesh, and Gujarat each reported 1 case.

**Figure 8:**
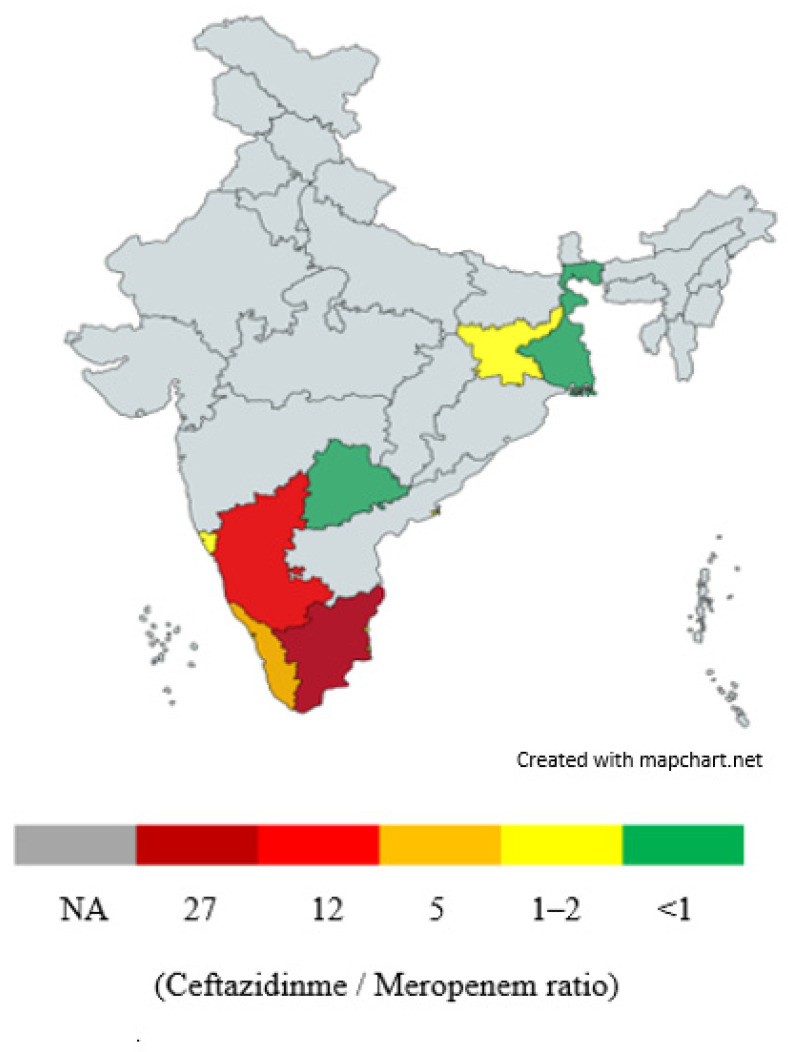
Ratio of ceftazidime/meropenem consumed by melioidosis patients in selected Indian states (1991–2018) (higher ratio shows preference for ceftazidime over meropenem, as seen in Tamil Nadu > Karnataka > Kerala > Odisha > Goa >Telangana > West Bengal).

Furthermore, as seen in Figure 8, the ratio of ceftazidime to meropenem consumption among melioidosis patients, as documented by Mukhopadhyay et al. [172], indicates a preference for ceftazidime over meropenem in the following states, ranked from highest to lowest preference: Tamil Nadu, Karnataka, Kerala, Odisha, Goa, Telangana and West Bengal.

### 3.5. Family Inclusive Capacity Building and Unconventional Approaches

The FIRE approach—inclusion of friends, relatives, and even enemies in capacity building—emerges as a novel strategy to address melioidosis, drawing inspiration from the societal response to the COVID-19 pandemic. Just as the pandemic made even the most uneducated individuals aware of basic infection control measures, including mask wearing, the same model was applied to build awareness and capacity for melioidosis prevention and management. The involvement of the entire community, beyond just medical professionals, played a crucial role in handling the COVID-19 crisis. Similarly, this broader approach could be utilized for melioidosis capacity building, ensuring that not only clinicians but also the general public play an active role in understanding and mitigating the disease (FIRE Approach to Capacity Building: Inclusion of Family and Friends, Relatives, even Enemies in Melioidosis Awareness) [66,67,68,69]

In the Indian context, the MISSION project, led by Kasturba Medical College (KMC) and funded by ICMR, aims to spread awareness and build capacity in melioidosis across 15 centers nationwide with a hub-and-spoke model, effectively linking primary and community healthcare to tertiary care centers. This strategic framework facilitated improved diagnostic capabilities, enabling timely identification of melioidosis cases across diverse regions, particularly in the eastern, northeastern, northern, and northwestern states of India, by equipping laboratories in tertiary care facilities and fostering collaboration with local healthcare providers. The initiative successfully diagnosed over 150 cases of melioidosis in two years. This comprehensive approach not only streamlined diagnostic processes but also significantly contributed to patient outcomes, demonstrating the critical importance of strengthening healthcare networks for better disease management and prevention. The project underscores the potential of integrated healthcare systems in saving lives through early detection and intervention. In addition to conventional capacity-building programs targeting healthcare professionals, the project has also incorporated family-inclusive approaches. For instance, a few staff members involved in the melioidosis project were inducted into understanding the disease through informal settings like social dinners with scientists. This inclusive model engaged individuals from various professional backgrounds, including school teachers and village officers. These efforts targeted vulnerable groups, such as children and farmers, who may be at higher risk due to their environmental exposures.

Through interactions with school teachers, insights were gained on how teaching methods during the COVID-19 pandemic prevented fomite transmission. Similarly, village officers, who govern soil transport policies within districts, were made aware of melioidosis hotspots, enabling them to design preventive measures for areas with a history of infection. Suggested policy measures derived from these discussions include:

- The use of computer-based systems for correcting test papers in schools to reduce fomite transmission.
- Training school teachers to recognize skin lesions associated with melioidosis, allowing them to identify potential cases in their classrooms.
- Informing village officers about melioidosis-positive zones to prevent the transport of soil from these areas to regions with negative testing results (Impact of school practices in preventing fomite transmission during COVID-19) [175].

Moreover, the integration of agricultural technologies such as soil health cards, could aid in identifying soil with parameters conducive to harboring *B. pseudomallei*. By leveraging these tools, the concerned authorities could proactively monitor and manage areas at higher risk, ultimately reducing the spread of the disease (Soil Health Card Scheme, Department of Agriculture and Government of India).

This multifaceted, community-driven approach not only strengthens awareness but also empowers various societal groups to contribute to the prevention of melioidosis, making capacity building a truly collective effort.

### 3.6. Recommendations and Future Directions

This review highlights several areas that need further investigation for better control of melioidosis in India. Although the ICMR-funded project initiated in 2022 by *KMC* across 14 states, including all 8 northeastern states, has made strides, the overall prevalence of melioidosis in many other states across India remains underexplored. Therefore, a targeted approach focusing on known risk factors such as diabetes and poverty, could be beneficial. Identifying states and districts with a high prevalence of these risk factors may provide a more effective strategy for melioidosis surveillance and control. Future studies should continue to examine the correlation between diabetes, poverty, and pneumonia with melioidosis risk, particularly in northeast India and districts with higher diabetes prevalence [70,176,177,178]. Hospitals that have published research on melioidosis are listed in Table 4 [179,180,181,182,183,184,185,186,187,188,189,190,191,192,193,194,195,196,197,198].

This suggests that future studies should focus on expanding data collection in these high-risk areas, as well as improving the integration of local hospitals and research initiatives into nationwide melioidosis control programs.

For microbiologists, it is essential to advance diagnostic capabilities for melioidosis, ensuring quicker, accurate and cost-effective testing, especially in under-resourced regions. Strengthening surveillance systems and diagnostic networks in high-risk areas is necessary for early detection and timely treatment.

Clinicians should be vigilant in recognizing melioidosis, particularly in regions with known risk factors such as diabetes and poverty. As melioidosis can present with symptoms similar to pneumonia or sepsis, early clinical suspicion in high-prevalence zones is crucial. Training and awareness programs about the disease’s clinical manifestations and management should be a priority for healthcare professionals.

Public health personnel and epidemiologists need to focus on identifying districts with a high prevalence of diabetes and poverty, as these are known risk factors for melioidosis. According to NFHS-5 data from 2019–2021, states with the highest incidence of diabetes include Kerala, Delhi and Ladakh, followed by Andhra Pradesh and Telangana. Other states with significant diabetes prevalence are Maharashtra, Tamil Nadu, Odisha, West Bengal, Arunachal Pradesh, Tripura, Jammu and Himachal Pradesh. These regions may therefore have a higher incidence of melioidosis, with high diabetes prevalence. Targeted public health strategies should be implemented in these areas to improve surveillance, prevention and control measures.

Government officials and policymakers must allocate resources to expand the ICMR’s efforts, focusing on high-risk states and districts and ensuring better integration of local hospitals and research centers into national melioidosis control programs. Future studies should continue to explore the link between diabetes, poverty and pneumonia with melioidosis, particularly in northeast India, to refine prevention and intervention strategies.

## 4. Discussion

Over the past two decades, the increasing number of melioidosis publications in India reflects growing awareness of the disease, particularly in south and eastern regions. However, there is still a need for more research in central, north and northeast India. Clinical studies indicate that diabetes is the most prevalent risk factor, with common symptoms such as fever, cough and joint pain. Septicemia and pneumonia are frequently observed in patients. These findings align with the global trends, but environmental factors like soil and water exposure, particularly following rainfall, play a significant role in India. There is also growing concern about the urban transmission of *B. pseudomallei*.

In India, while specific data comparing the use of ceftazidime and carbapenems for treating melioidosis are limited, studies from Indian tertiary care centers, particularly in South India, provide valuable insights. These studies show that nearly 100% of *B. pseudomallei* isolates are sensitive to both ceftazidime and imipenem, with the rare exception of ceftazidime-resistant *B. pseudomallei* masquerading as isolated atypical neuro melioidosis [162]. This high sensitivity to ceftazidime supports its potential as a cost-effective alternative to carbapenems in the LMICs like India, which are more expensive. While the studies do not directly indicate that ceftazidime is more commonly used due to cost considerations, the effectiveness of ceftazidime in treating melioidosis is evident. The favourable sensitivity rates and outcomes associated with ceftazidime suggest it could be a viable option for treatment in resource-limited settings, such as India. Despite these promising findings, more research is needed to confirm the comparative efficacy and cost-effectiveness of ceftazidime versus carbapenems for treating melioidosis, ensuring that clinical decisions are informed by both efficacy and economic factors.

Further, the correlation between diabetes prevalence and melioidosis risk suggests that states with higher diabetes rates, such as Kerala and Tamil Nadu, may be at greater risk, warranting targeted public health interventions.

Moreover, the FIRE approach, focusing on community involvement for capacity building, offers an innovative model for raising awareness and preventing melioidosis, particularly among vulnerable populations. Future research should focus on environmental surveillance, outbreak monitoring, alternative treatments, and the effectiveness of community-based interventions to reduce the impact of melioidosis in India. These efforts will be crucial in mitigating the disease burden on the population and in improving overall public health strategies.

Melioidosis is often under-diagnosed due to its nonspecific symptoms, making surveillance crucial. The authors propose the following surveillance and intervention strategies for melioidosis in India, which should focus on strengthening early detection, public awareness and targeted interventions:

- **Enhanced Surveillance:** Establish a national melioidosis surveillance network to report cases from hospitals, particularly in endemic regions like northeast India. Regular screening in high-risk populations, including those with diabetes, CLD, and immune-compromised conditions, is essential. Laboratories should be equipped with advanced diagnostic tools like PCR and culture techniques. National programs similar to those for AIDS and viral hepatitis could help in integrating *B. pseudomallei* testing with India’s agricultural soil portal and promoting community awareness of hygiene practices to reduce infection risks. Erythritol, often used by diabetic patients as a sugar alternative, is utilized in lab cultivation of *B. pseudomallei*, necessitating surveillance studies on diabetic melioidosis patients for better intervention.
- **Healthcare Provider Training:** Educate healthcare professionals about melioidosis’ clinical presentation, diagnostic methods and treatment protocols. Early recognition is a key to reducing mortality.
- **Public Awareness Campaigns:** Launch campaigns to raise awareness among the general public, particularly in areas with poor sanitation and high agricultural activity. Emphasize preventive measures such as avoiding contaminated water and soil.
- **Environmental Control:** Address environmental risk factors by improving water management and sanitation in high-risk areas, especially rural and agricultural zones. Implementing practices like proper handling of soil and water in farming can help reduce exposure to *B. pseudomallei*. Develop community awareness and enact environmental sampling from water bodies used for traditional practices like ritual scalp shaving and ceremonies, as these are shown to be risk factors for sepsis [199]. Further practicing the same during birth and death rituals near ponds, lakes and rivers could address an important risk factor for pneumonia and sepsis. Attempt the integration of governmental bodies, such as Food Safety, Central Pollution Control Board, Drinking Water & Sanitation and so on, in monitoring food products and water bodies, respectively, that tend to pose a risk of harboring *B. pseudomallei.*
- **Research Collaboration and Coordination:** Support research on melioidosis epidemiology, diagnostics and treatment. Implementation of food safety regulation to sell soil-free vegetables could minimize the load of agricultural soil entering poor and middle-income households. In addition, collaborate with international health organizations for expertise and funding.
- **Adapting strategies from other Endemic Countries:** In endemic countries like Thailand and Australia, control measures for melioidosis focus on raising awareness about infection risks through contaminated soil and water and promoting the use of protective gear. In rural areas of Australia, patients are swiftly transferred to referral hospitals for antibiotic treatment. Vaccination may be planned for high-risk groups.

## 5. Conclusions

Melioidosis is emerging as a significant public health threat in India, with increasing recognition of its impact as a tropical killer disease. This review not only highlights the rising volume of research and publications focused on melioidosis in the country, but also points to critical gaps in understanding and response, especially in areas such as environmental surveillance, vaccines and diagnostic development. It also addresses regional disparities in disease awareness and detection. While South and Eastern India show higher awareness and detection rates, other regions, particularly Northeast India, continue to lag, underscoring the need for more targeted interventions. Prevention strategies focusing on minimizing exposure, especially for high-risk groups such as farmers and those with underlying health conditions, together with public awareness campaigns (emphasizing the importance of wearing protective clothing and avoiding contact with contaminated soil and water), can help reduce the infection rates.

The economic burden of melioidosis is also a pressing concern, particularly due to the out-of-pocket costs associated with antibiotics treatment, which involves the use of ceftazidime, being the first-line therapy. However, use of such cheaper alternatives over more expensive drugs like carbapenems may contribute to the rise of drug resistance, which necessitates ongoing surveillance and research into alternative treatment options. In addition, post-treatment follow-up is essential, as relapses can occur. Strengthening healthcare infrastructure, enhancing laboratory capabilities and promoting research will be vital in addressing melioidosis effectively in India.

This reflects the complex interaction of socio-economic factors in treatment choices and health outcomes. In regions without clear hotspots, using risk factors such as diabetes and poverty as presumptive markers could aid in early detection and intervention.

Furthermore, the lack of research on vaccines and diagnostic tools remains a major challenge in improving patient care. Early diagnosis is critical, as melioidosis can present with a wide range of symptoms, often mimicking other diseases. Health professionals should maintain a high index of suspicion, especially in patients with pneumonia or septicemia, and consider travel history to endemic areas. Utilizing advanced diagnostic techniques, such as PCR and culture methods, can facilitate timely identification of the pathogen. Integration of IVD kits that have dual detection for melioidosis with other diseases like dengue, hepatitis, malaria or tuberculosis could promote wider population coverage. Further, cryptic environmental factors like blow flies and erythritol could be further researched in the context of the environmental presence of *B. pseudomallei*. Environmental spillage estimation of *B. pseudomallei* at stray animal accident sites with improper carcass disposal could be attempted.

Innovative strategies, such as leveraging soil health cards and family-based initiatives, offer potential pathways for increasing awareness and community engagement in melioidosis control. By adopting these strategies and learning from global experiences, India can better address this emerging disease and reduce its impact on public health. The growing burden of melioidosis underscores the need for urgent, coordinated action to safeguard the nation’s health.

## Figures and Tables

**Table 3:** Clinical profile reported in the literature.

Studies		*N* = 7 [131]	*N* = 19 [12]	*N* = 26 [90]	*N* = 21 [90]	*N* = 73 [66]	*N* = 114 [128]	*N* = 37 [63]	*N* = 58 [63]	*N* = 41 [68]	*N* = 25 [69]
**Location**		**Central India**	**South India**	**East India**	**East India**	**South India**	**South India**	**South India**	**South India**	**South India**	**South India**
	**Clinical** **Manifestation**										
Fever		100%	89%	100%	67%	–	97%	–	–	83%	80%
Cough		–	42%	23%	5%	–	16%	–	–	46%	–
Joint Pain		100%	37%	4%	0%	–	25%	–	–	–	48%
Abscess		–	16%	–	–	–	–	–	–	–	–
Skin and Soft Tissue/Cutaneous		–	11%	–	–	25%	13%	–	3.2%	27%	–
Bacteremia			58%	100%	0%	34%	55%	100%	0%	54%	
Skin and Soft tissue/Cutaneous		–	11%	–	–	25%	13%	–	3.2%	27%	–
Septic Arthritis/Arthritis		–	11%	4%	–	12%	19%	5.3%	10.5%	–	–
Pneumonia/Respiratory Involvement		71%	5%	–	–	41%	–	54%	12.6%	61%	48%
Splenic Abscess/Involvement of Spleen		–	5%	8%	–	40%	43%	2.1%	2.1%	22%	24%
**Location**		**Central India**	**South India**	**East India**	**East India**	**South India**	**South India**	**South India**	**South India**	**South India**	**South India**
	**Risk** **Factors**										
Diabetes		100%	84%	–	–	72%	82%	62.2%	–	79%	68%
Hypertension		–	–	–	–	32%	–	–	–	–	–
CKD		–	10%	–	–	10%	4%	24.3%	–	–	–
COPD		–	10%	–	–	–	–	–	–	–	–
Liver Disease		–	10%	–	–	–	–	–	–	–	–
Alcohol		–	–	–	–	–	14%	8.1%	–	24%	28%
Tuberculosis		–	5%	–	–	–	–	–	–	–	–
Cancer		–	5%	–	–	7%	–	–	–	–	–
No Focus		–	5%	–	–	–	–	–	–	–	–

**Table 4:** Hospitals in specified Indian states that have publications on melioidosis [7,179,180,181,182,183,184,185,186,187,188,189,190,191,192,193,194,195,196,197,198].

**Kerala**
Kerala Institute of Medical Sciences (KIMS), Trivandrum
Department of Neurology, Sree Chitra Tirunal Institute for Medical Sciences and Technology, Trivandrum
BMH Gimcare Hospital, Kannur
St. James Hospital, Chalakudy
Department of Orthopaedics, Government Medical College, Kozhikode
Government Medical College, Thiruvananthapuram
Department of Neurosurgery, Lisie Hospital, Ernakulam
**Karnataka**
Department of Microbiology, Father Muller Medical College, Mangalore
Department of Medicine, MVJMC and RH, Hoskote, Bengaluru
Department of Pulmonary Medicine, K.S. Hegde Medical Academy, Mangaluru
**Odisha**
Department of General Medicine, Kalinga Institute of Medical Sciences, Bhubaneswar
Department of Microbiology, All India Institute of Medical Sciences, Bhubaneswar
Internal Medicine, Srirama Chandra Bhanja (SCB) Medical College and Hospital, Cuttack
Department of Microbiology, Kalinga Institute of Medical Sciences, Bhubaneswar
**Rajasthan**
Fortis Escorts Hospital, Jaipur
Department of Medicine, All India Institute of Medical Sciences, Jodhpur
**Assam**
ICU and Critical Care, Ayursundra Super Speciality Hospital, Guwahati
Department of Microbiology, Excelcare Hospitals, Guwahati
Department of Medicine, GMCH, Guwahati, Kamrup (Metro)
**Delhi**
Department of Microbiology, Fortis Flt. Rajan Dhall Hospital, Vasant Kunj
Neurology, AIIMS
Department of Medicine and Microbiology, Army Hospital (Research and Referral)
Department of General Medicine, Sir Ganga Ram Hospital

## Data Availability

Data are contained within the article and Supplementary Materials.

## Supplementary Materials

The following supporting information can be downloaded at: https://www.mdpi.com/article/10.3390/pathogens14040379/s1, Table S1: Year-wise publications and grouping based on study site, purpose, study type, data and authors. Articles retrieved from PubMed and Science Direct as per methods detailed in Figure 1a with search criteria–“Melioidosis prevalence India”; Table S2: Year-wise publications and grouping based on study site, purpose, study type, data and authors. Articles retrieved from PubMed and Science Direct as per methods detailed in Figure 1b with search criteria—“Melioidosis risk factor India”.
