# Supplementary material for: Two Decades of Melioidosis in India: A Comprehensive Epidemiological Review
Source: Pathogens. 2025 Apr 14;14(4):379. doi: 10.3390/pathogens14040379 (PMC12030058; doi:10.3390/pathogens14040379)
Supplement: Supplementary file 1 [file pathogens-14-00379-s001.zip › pathogens-3476178-supplementary.pdf]

S.No	Year of publication (20XX)	South India	West India / South West	North India	East India	Central India	Environmental surveillance	Clinical	Outbreak Surveillance	Risk factors	Longitudinal study	Cross sectional study	Reviews / Research / Epidemiology	Case series / Hospital based study	Prevalence reporting	Retrospective cohort	Drugs	Vaccine	Development of diagnostics	Novel therapy	Medical practice	General (lab diagnosis)	National	International
**Total**		50	18	7	9	4	7	62	3	4	1	0	26	33	7	4	2	0	10	1	52	23	66	9
Search in PubMed																								
1	24	Y						Y								Y					Y		Y	
2	24	Y						Y								Y					Y		Y	
3	24	Y						Y						Y							Y		Y	Y
4	24	Y							Y	Y				Y								Y	Y	
5	24	Y						Y		Y											Y		Y	Y
6	24		Y						Y					Y								Y	Y	
7	24	Y						Y								Y			Y		Y		Y	
8	24	Y						Y						Y					Y		Y		Y	Y
9	23							Y					Y						Y		Y		Y	
10	23			Y				Y											Y		Y		Y	
11	22				Y			Y						Y							Y		Y	
12	22	Y						Y					Y				Y			Y			Y	Y
13	22				Y			Y							Y						Y		Y	
14	22	Y					Y				Y											Y	Y	
15	22	Y					Y	Y						Y							Y		Y	
16	22				Y			Y						Y							Y		Y	
17	21	Y						Y						Y							Y		Y	
18	21			Y				Y						Y							Y		Y	
19	21	Y						Y					Y								Y		Y	
20	21	Y						Y								Y					Y		Y	
21	21				Y			Y						Y					Y			Y	Y	
22	21		Y	Y				Y					Y								Y		Y	
23	21				Y			Y					Y								Y		Y	
24	21	Y						Y						Y							Y		Y	
25	21			Y				Y					Y								Y		Y	
26	20	Y						Y						Y							Y	Y	Y	
27	19				Y			Y					Y								Y	Y	Y	
28	19	Y						Y						Y							Y		Y	
29	19	Y					Y	Y					Y								Y		Y	
30	19	Y						Y					Y				Y				Y		Y	
31	18	Y						Y					Y	Y							Y		Y	
32	18	Y	Y					Y						Y								Y	Y	
33	18	Y				Y		Y						Y							Y	Y	Y	
34	18	Y						Y					Y	Y							Y	Y	Y	
35	18	Y					Y	Y					Y									Y	Y	

36	17	Y	Y					Y						Y							Y		Y	
37	17	Y	Y					Y						Y							Y		Y	
38	16	Y						Y					Y									Y	Y	
39	16				Y			Y						Y							Y		Y	
40	16	Y	Y				Y						Y									Y	Y	
41	16	Y	Y					Y							Y							Y	Y	
42	16									Y			Y								Y			Y
43	14							Y					Y								Y			Y
44	14	Y				Y	Y								Y							Y	Y	
45	13	Y						Y					Y								Y		Y	
46	13	Y	Y					Y						Y							Y		Y	
47	12	Y						Y						Y					Y			Y	Y	
48	12		Y			Y		Y					Y								Y		Y	
49	10	Y	Y					Y					Y								Y		Y	
50	10	Y	Y					Y							Y						Y		Y	
51	10	Y	Y					Y						Y							Y		Y	
52	10	Y						Y					Y						Y		Y	Y	Y	
53	O8									Y					Y						Y			Y
54	O8	Y	Y					Y							Y							Y	Y	Y
55	O8	Y	Y					Y						Y							Y		Y	
56	O8	Y	Y					Y						Y							Y	Y	Y	
57	O8	Y						Y						Y							Y		Y	
58	O7	Y	Y					Y						Y	Y							Y	Y	
59	O7	Y					Y		Y				Y									Y		Y
60	O3	Y						Y						Y							Y		Y	
61	O1	Y						Y					Y						Y			Y	Y	
Search in Science Direct																								
62	22				Y			Y					Y								Y		Y	
63	23			Y				Y						Y							Y		Y	
64	24	Y						Y						Y							Y		Y	
65	24	Y						Y					Y								Y		Y	
66	18	Y	Y	Y	Y			Y					Y						Y			Y	Y	
67	18	Y						Y						Y							Y		Y	
68	20	Y	Y					Y						Y							Y		Y	
69	13	Y						Y						Y							Y		Y	
70	15			Y		Y		Y					Y						Y			Y	Y	

Supplementary Table S1 Year wise publications and grouping based on study site, study type, authors. Articles retrieved from PubMed (S. No:1-61) and Science Direct (S. No:62-70) as per methods detailed in Figure 1a with search criteria – "Melioidosis prevalence India". "Y" refers to Yes in the table. [1,7,27,32,40,63-126]

Table S2

S.No	Year of Publication (20XX)	South India	West India / South West	North India	East India	Central India	Environmental surveillance	Clinical	Outbreak Surveillance	Risk factors	Longitudinal study	Cross sectional study	Reviews / Research / Epidemiology	Case series / Hospital based study	Prevalence reporting	Retrospective cohort	Drugs	Vaccine	Development of diagnostics	Novel therapy	Medical practice	General (lab diagnosis)	National	International
Total		43	21	6	3	1	2	55	0	5	0	0	20	36	2	2	4	2	4	0	49	12	56	8
**Search in Pub Med**																								
1	24	Y	Y					Y					Y	Y							Y		Y	Y
2	24	Y	Y					Y						Y			Y					Y	Y	
3	23					Y		Y					Y								Y	Y	Y	
4	23	Y						Y						Y							Y		Y	
5	22	Y	Y							Y			Y								Y		Y	
6	22	Y						Y						Y					Y			Y	Y	
7	21	Y						Y					Y								Y		Y	
8	21	Y						Y					Y								Y		Y	
9	21	Y						Y		Y			Y								Y		Y	Y
10	21	Y						Y						Y							Y		Y	
11	21				Y			Y						Y					Y			Y	Y	
12	21	Y						Y					Y	Y							Y		Y	
13	20	Y	Y					Y					Y								Y		Y	Y
14	20	Y	Y					Y					Y								Y	Y	Y	
15	19	Y						Y						Y							Y		Y	
16	19		Y				Y	Y						Y							Y		Y	Y
17	19	Y	Y				Y	Y						Y							Y	Y	Y	
18	19	Y						Y								Y					Y		Y	
19	19	Y						Y						Y			Y				Y		Y	
20	19	Y						Y						Y							Y		Y	
21	19	Y						Y						Y							Y		Y	
22	18	Y						Y								Y					Y		Y	
23	16				Y			Y						Y							Y		Y	
24	16	Y	Y					Y							Y						Y		Y	
25	16									Y					Y						Y			Y
26	14			Y				Y						Y							Y		Y	
27	13	Y						Y					Y								Y		Y	
28	14	Y	Y					Y						Y							Y		Y	
29	13	Y						Y						Y							Y		Y	
30	13	Y						Y						Y							Y		Y	
31	10	Y	Y					Y						Y							Y		Y	

32	10	Y						Y						Y							Y		Y	
33	o9	Y	Y					Y						Y							Y		Y	
34	o8	Y						Y						Y							Y		Y	
35	o7			Y				Y						Y							Y		Y	
**Search in Science Direct**																								
36	24	Y	Y	Y				Y						Y					Y			Y	Y	Y
37	24		Y	Y				Y						Y							Y		Y	
38	23	Y						Y						Y							Y		Y	
39	24	Y						Y		Y				Y							Y		Y	
40	23				Y			Y		Y				Y							Y		Y	
41	21	Y	Y					Y						Y							Y		Y	
42	19	Y						Y						Y			Y						Y	
43	23		Y					Y						Y			Y					Y	Y	
44	22		Y					Y						Y							Y		Y	
45	24	Y						Y						Y							Y		Y	
46	19	Y	Y					Y					Y					Y					Y	
47	24			Y				Y						Y							Y		Y	
48	14			Y				Y					Y					Y				Y	Y	
49	16	Y						Y					Y								Y	Y	Y	
50	**16**		Y					Y					Y								Y		Y	
51	16	Y						Y					Y								Y		Y	
52	14	Y	Y					Y					Y								Y		Y	
53	o8	Y						Y					Y								Y		Y	
54	o7	Y						Y					Y								Y		Y	
55	24	Y	Y					Y					Y	Y							Y		Y	Y
56	24	Y	Y					Y					Y								Y	Y	Y	Y
57	18	Y						Y						Y					Y		Y	Y	Y	

Supplementary Table S2 Year wise publications and grouping based on study site, study type, authors. Articles retrieved from PubMed (S. No:1-35) and Science Direct (S. No: 36-57) as per methods detailed in Figure 1b with search criteria – "Melioidosis risk factor India". "Y" refers to Yes in the table. [11,12,27,33,49,66-69,127-171].
